# A randomised phase II multicentre trial of irinotecan (CPT-11) using four different schedules in patients with metastatic colorectal cancer

**DOI:** 10.1038/sj.bjc.6602172

**Published:** 2004-09-21

**Authors:** N E Schoemaker, I E L M Kuppens, V Moiseyenko, B Glimelius, M Kjaer, H Starkhammer, D J Richel, R Smaaland, K Bertelsen, J P Poulsen, E Voznyi, J Norum, D Fennelly, K M Tveit, A Garin, G Gruia, A Mourier, D Sibaud, P Lefebvre, J H Beijnen, J H M Schellens, W W ten Bokkel Huinink

**Affiliations:** 1 Antoni van Leeuwenhoek Hospital/The Netherlands Cancer Institute, Amsterdam, The Netherlands; 2 Slotervaart Hospital, Amsterdam, The Netherlands; 3 Petrov Research Institute of Oncology, St-Petersburg, Russia; 4 Onkologiska Kliniken, Uppsala, Sweden; 5 Aalborg Hospital, Aalborg, Denmark; 6 Onkologiska Kliniken, Linkoping, Sweden; 7 Medisch Spectrum Twente, Enschede, The Netherlands; 8 Haukeland Hospital, Bergen, Norway; 9 Odense University Hospital, Odense, Denmark; 10 The Norwegian Radium Hospital, Oslo, Norway; 11 Research Institute of Diagnostic and Surgery, Moscow, Russia; 12 The Regional Hospital of Tromso, Tromso, Norway; 13 St Vincent Hospital, Dublin, Ireland; 14 Ullevaal University Hospital, Oslo, Norway; 15 All-Union Cancer Research Center, Moscow, Russia; 16 Aventis Pharma, Antony Cedex, France; 17 Utrecht University, Faculty of Pharmaceutical Sciences, The Netherlands

**Keywords:** colorectal cancer, CPT-11, efficacy, irinotecan, pharmacokinetics, safety

## Abstract

The purpose of this phase II trial was to compare the efficacy, safety and pharmacokinetics of four irinotecan schedules for the treatment of metastatic colorectal cancer. In total, 174 5-fluorouracil pretreated patients were randomised to: arm A (*n*=41), 350 mg m^−2^ irinotecan as a 90-min i.v. infusion q3 weeks; arm B (*n*=38), 125 mg m^−2^ irinotecan as a 90-min i.v. infusion weekly × 4 weeks q6 weeks; arm C (*n*=46), 250 mg m^−2^ irinotecan as a 90-min i.v. infusion q2 weeks; or arm D (*n*=49), 10 mg m^−2^ day^−1^ irinotecan as a 14-day continuous infusion q3 weeks. No significant differences in efficacy across the four arms were observed, although a shorter time to treatment failure was noted for arm D (1.7 months; *P*=0.02). Overall response rates were in the range 5–11%. Secondary end points included median survival (6.4–9.4 months), and time to progression (2.7–3.8 months) and treatment failure (1.7–3.2 months). Similarly, there were no significant differences in the incidence of grade 3–4 toxicities, although the toxicity profile between arms A, B, and C and D did differ. Generally, significantly less haematologic toxicity, alopecia and cholinergic syndrome were observed in arm D; however, there was a trend for increased gastrointestinal toxicity. Irinotecan is an effective and safe second-line treatment for colorectal cancer. The schedules examined yielded equivalent results, indicating that there is no advantage of the prolonged *vs* short infusion schedules.

Irinotecan (CPT-11, Campto®) is a semisynthetic water-soluble derivative of the plant alkaloid camptothecin. Irinotecan is used for the first- and second-line treatment of advanced colorectal cancer following 5-fluorouracil (5-FU)-based therapy (Cunningham *et al*, 1998; Douillard *et al*, 2000). Unlike other camptothecin analogues, irinotecan is a prodrug and undergoes hydrolysis by liver carboxylesterase to form the active metabolite SN-38 (Rivory *et al*, 1996). Irinotecan specifically stabilises covalent topoisomerase I (TopI)–DNA cleavable complexes, which ultimately leads to cell death (Hsiang *et al*, 1985, 1989a; Bjornsti *et al*, 1989). The formation of the TopI-cleavable complex is reversible; it exists only in the presence of a camptothecin-like drug (Bjornsti *et al*, 1989; Stewart *et al*, 1998). The cytotoxic effect of irinotecan and SN-38 is cell cycle specific and therefore prolonged infusions could increase the antitumour activity (Hsiang *et al*, 1989b; Burris *et al*, 1992).

Preclinical data and some clinical data support the use of prolonged exposure schedules of camptothecins (Furuta and Yokokura, 1990; Houghton *et al*, 1995; O'Reilly and Rowinsky, 1996). Phase I studies have employed different administration schedules and dosages of irinotecan, including a prolonged infusion schedule. Diarrhoea and/or neutropenia were the major dose-limiting toxicities in these studies (Masuda *et al*, 1996; O'Reilly and Rowinsky, 1996; Herben *et al*, 1999). Three schedules with short infusions were used in phase II studies, a 90-min infusion once every 3 weeks, a 90-min weekly infusion and a fortnightly 90-min infusion (Shimada *et al*, 1993; Rothenberg *et al*, 1996; Pitot *et al*, 1997; Rougier *et al*, 1997; Cunningham *et al*, 1998; Douillard *et al*, 2000). In order to further evaluate the risk–benefit ratio of irinotecan (after failure of thymidylate synthase inhibitor-based regimens, for example, 5-FU) at the currently recommended dosages/schedules in patients with metastatic colorectal cancer and to determine whether a clinically relevant schedule dependency exists for irinotecan, this multicentre, randomised, open-label phase II study was initiated. Patients were randomised to one of four treatment arms: 350 mg m^−2^ irinotecan q3 weeks (arm A), 125 mg m^−2^ irinotecan weekly × 4 weeks q6 weeks (arm B), 250 mg m^−2^ irinotecan q2 weeks (arm C) and a 14-day continuous infusion of 10 mg m^−2^ day^−1^ q3 weeks (arm D). The primary objective of the study was to determine and rank the response rates of the four different irinotecan schedules. The secondary objectives were to determine the time to progression and treatment failure, duration of response and disease stabilisation, and survival. Furthermore, pharmacokinetics were evaluated for arms A, B and C, and a possible schedule dependency for irinotecan was explored.

## MATERIALS AND METHODS

### Inclusion/exclusion criteria

Patients were included in the study if they had histologically proven adenocarcinoma of the colon or rectum and metastatic disease at study entry (within 6 months of the last thymidylate synthase inhibitor administration). Only patients with measurable metastatic disease were included, and if only one metastatic lesion was present a histologic examination was mandatory. Other inclusion criteria included: age 18–75 years, World Health Organization (WHO) performance status (PS) ⩽2, and estimated life expectancy of >3 months (Anonymous, 1979). Patients had to have sufficient haematologic function, defined as neutrophil count ⩾1.0 × 10^9^ l^−1^, haemoglobin ⩾6.5 g dl^−1^ and platelet count ⩾50 × 10^9^ l^−1^; adequate hepatic and renal function, defined as total bilirubin ⩽1.5 times the upper normal limit (UNL), transaminases ⩽3 times UNL or ⩽5 times when related to liver metastases and serum creatinine ⩽1.5 times UNL.

All patients should have received adjuvant and/or palliative chemotherapy based on thymidylate synthase inhibitors (5-FU or raltitrexed) within 6 months of study entry. Patients could not have undergone chemotherapy or surgery within at least 4 weeks before entry into the study, or 6 weeks in the case of mitomycin C, nitrosourea or extended field radiation therapy. The overall number of prior chemotherapy regimens could not exceed 2, if one of them was given with adjuvant intent, or 1 if only a palliative regimen was given. Patients had to be suitable and willing to undergo insertion of a portable device and indwelling catheter (Port-a-Cath).

Exclusion criteria included a history of treatment with any topoisomerase inhibitor, chronic enteropathy (Crohn's disease, ulcerative colitis), bowel obstruction or sub-obstruction, prior or current history of chronic diarrhoea, symptomatic brain metastasis, current infection, other serious illness or medical conditions or a history of other cancer, except adequately treated *in situ* cervical carcinoma or nonmelanoma skin cancer. The study protocol was approved by an independent Ethics Committee, in agreement with local legal prescriptions. All patients gave written informed consent.

### Trial design

This was a prospective, nonblinded, multicentre, phase II study, which planned to comprise at least 148 eligible and evaluable patients. A total of 17 centres in Europe participated in this study: two in Denmark, one in Ireland, four in The Netherlands, five in Norway, two in Sweden and three in Russia. Patients were treated with four different schedules of irinotecan. Randomisation was performed centrally by Aventis Pharma R&D, Antony Cedex, France. The primary end point of this study was response rate; the Simon design was used to rank these results. A total of 37 evaluable patients per arm had to be included. With this sample size and the hypothesis that the lowest expected baseline response rate would be 10%, there was a 90% probability of selecting the best treatment group on categorical variables.

### Treatment plan

Patients randomised to arm A received 350 mg m^−2^ irinotecan as a 90-min i.v. infusion every 3 weeks, patients in arm B received 125 mg m^−2^ irinotecan as a 90-min i.v. infusion weekly for 4 weeks every 6 weeks and patients in arm C received 250 mg m^−2^ irinotecan as a 90-min infusion every 2 weeks. Patients in arm D were treated with irinotecan given as a 14-day infusion at a dose level of 10 mg m^−2^ day^−1^, which was repeated every 3 weeks. One treatment course was defined as one 90-min infusion in arms A, B or C (every 3 weeks, weekly or every 2 weeks, respectively) and a continuous infusion over 14 days administered every 3 weeks in arm D. A cycle was defined as a treatment period of 6 weeks; a complete cycle for patients in arms A and D consisted of two courses of irinotecan, yielding total dose intensities of 700 and 280 mg m^−2^ per 6 weeks, respectively, a cycle in arm B consisted of four courses yielding a dose intensity of 500 mg m^−2^ per 6 weeks and a cycle for patients in arm C consisted of three courses of irinotecan, yielding a dose intensity of 750 mg m^−2^ per 6 weeks.

### Toxicity and response evaluation

Pretreatment evaluation included a complete medical history and complete physical examination. Before each course, blood chemistry and haematology profiles were checked. Haematology was checked weekly. Tumour measurements were performed every other cycle (6 weeks) and responses were scored according to WHO criteria (Anonymous, 1979). An external response review committee (ERRC) was established during the study to perform assessments of tumour response. All toxicities were graded according to the National Cancer Institute-Common Toxicity Criteria (NCI-CTC) (Anonymous, 2004). Adverse events that were not reported according to NCI-CTC were graded as mild, moderate, severe, or life threatening.

### Pharmacokinetics

A pharmacokinetic evaluation was performed using a population approach; 88 patients from arms A, B and C were evaluated during the first course of treatment. The sampling strategy consisted of three different sampling schedules (with three sampling times in each): schedule 1: 30 min before the end of the infusion, 5 min and 3–4 h post-infusion; schedule 2: 30 min before the end of the infusion, 10 min and 5–7 h post-infusion; schedule 3: 5 min before the end of the infusion, 30 min and 20–24 h post-infusion. In a few cases, an additional sample was taken in schedules 1 and 2 after 24 h. All patients at one site were sampled according to the same schedule. The aim of the sampling strategy was to define the full kinetic profile over the whole population, by drawing a small number of samples at different times from a large number of patients. This approach is previously referred to as the full-screen approach (Sheiner and Benet, 1985).

Blood samples (5 ml) were collected in heparinised tubes and immediately immersed in ice-water. Plasma was obtained by centrifugation of the samples (5 min; 3000 r.p.m., 4°C) and stored at −20°C or lower until analysis. Plasma levels of total (lactone plus carboxylate) irinotecan and metabolite SN-38 were determined by a reversed-phase high-performance liquid chromatography method using a fluorescence detection (Vergniol *et al*, 2000). The bio-analysis was performed in accordance with the principles of Good Laboratory Practice (Anonymous, 2001). The quantification limits for irinotecan and metabolite SN-38 were 10 and 2.5 *μ*g l^−1^, respectively. The plasma concentration–time profiles of irinotecan and metabolite SN-38 were analysed using nonlinear mixed effect modelling as implemented in the NONMEM program. Pharmacokinetic parameters were determined by the Bayesian approach with the concentration–time data from each patient, and a population model previously defined. A three- and two-compartment structural model were used for irinotecan and metabolite SN-38, respectively. The following pharmacokinetic parameters of irinotecan and SN-38 were determined: maximum plasma concentration (*C*_max_), time to reach *C*_max_ (*T*_max_), area under the plasma concentration–time curve from 0 to infinity (AUC) and total body clearance (CL, irinotecan only) was also estimated. The metabolic ratio of irinotecan was defined as the ratio of the SN-38 AUC over the irinotecan AUC. Furthermore, the AUC of SN-38 per cycle (AUC_c_) was estimated by multiplying the AUC of SN-38 by the number of courses per cycle (× 2, × 4 and × 3 for arms A, B and C, respectively).

### Statistical analysis

The primary efficacy variable was response rate; overall response was defined as complete plus partial response. The secondary efficacy variables were the duration of response and stabilisation, time to progression, time to treatment failure and the overall survival. The differences between the four treatment groups were explored with the *χ*^2^ test. Subsequent statistical tests were performed only in the case of evidence of difference to provide the significance level of difference. The Fisher exact test was used to compare treatment groups on categorical variables. For continuous variables, if a normal distribution could be assumed, Student's *t*-tests were used. Otherwise, they were analysed by nonparametric methods. Exact confidence intervals were calculated using binominal distribution probability. Censored data were analysed using the Kaplan–Meier method; the log-rank test was used to compare the groups. Differences in pharmacokinetic parameters between the four treatment arms were evaluated using one-way analysis of variance (ANOVA) combined with least significant difference (LSD) method (*P* was set at 0.05) and the Student's *t*-test was used to calculate the *P*-value of significant differences. The Pearson correlation coefficient (*r*) was calculated between dose and pharmacokinetic parameters. Statistical analyses were performed with SPSS (version 10.0.7 for Windows, SPSS Inc.). All tests for significance were two-tailed.

## RESULTS

### Patient enrollment and evaluability

A total of 174 patients entered the study. Of these patients, 168 (97%) actually received medication and comprised the intent to treat population (treated patients analysed in the arm to which they were assigned by randomisation). In all, 41 patients were included in arm A, 37 patients in arm B, 46 patients in arm C and 44 patients in arm D. A total of 149 (86%) patients were not in the per protocol population (treated, eligible and evaluable for response). Table 1

**Table 1:** Reasons for noneligibility or nonevaluability for response

**Reason^a^**	**Arm A**	**Arm B**	**Arm C**	**Arm D**
Presence or history of CNS metastasis		1	1	1
No measurable lesion	1			
Patient not metastatic		1		
Early discontinuation		3	1	8
Early death		1		
Response not properly assessed	1			
>1 line palliative chemotherapy		1		
Total bilirubin >1.5 UNL				1
ASAT and ALAT >5 UNL				1

a A patient may have more than one reason.

describes the reasons why patients were in the per protocol population. A total of 88 (51%) patients were evaluable for pharmacokinetics. At the cutoff date, all but seven patients had discontinued the treatment. In all treatment arms, the majority of patients discontinued treatment because of progressive disease: 81% in arm A, 57% in arm B, 61% in arm C and 55% in arm D. More patients discontinued treatment due to toxicity (from the study drug) in arm D (21%) compared with arm A (0%), arm B (14%) and arm C (11%) (*P*=0.03); the main reasons were diarrhoea in arm B (8%) and C (7%), diarrhoea (14%) and vomiting (14%) in arm D.

### Patient characteristics

Patient characteristics at baseline were well balanced across the four treatment arms and are summarised in Table 2

**Table 2:** Patient and disease characteristics

**Characteristics**	**Arm A**	**Arm B**	**Arm C**	**Arm D**
Number of patients	41	37	46	44
Male/female	23/18	17/20	27/19	22/22
Median age (range)	60 (28–75)	58 (41–71)	62 (35–74)	60 (31–76)
				
	**Percentage of patients**			
*WHO performance status*				
0	29	35	46	34
1	68	54	52	64
2	2	11	2	2
				
*Prior therapy*				
Surgery	100	97	96	100
Radiotherapy	29	19	24	14
Chemotherapy	2	14	7	9
				
*Primary tumour site*				
Colon	54	65	59	71
Rectum	46	35	41	30
	
*Number of involved organs*				
1	71	54	65	73
2	12	41	26	21
⩾3	17	5	9	7
	
*Involved organs*				
Liver	85	84	72	68
Lung	27	27	30	11
Lymph nodes	15	24	22	25
Soft tissue	5	0	9	18
Other	22	16	17	14

. The majority of patients had colon carcinoma. Diagnosis of the primary disease was made more than a year before the patients were randomised.

### Treatment delivery

A total of 247 courses were administered to 41 patients in arm A, 98 courses to 37 patients in arm B, 368 courses to 46 patients in arm C and 133 courses to 44 patients in arm D. All patients received at least one course. The median numbers of weeks on study were 18.6, 12.0, 15.2 and 7.0 in arms A, B, C and D, respectively. Details of treatment delivery and dose intensity are shown in Table 3

**Table 3:** Irinotecan treatment and extent of exposure

	**Arm A**	**Arm B**	**Arm C**	**Arm D**
Number of patients	41	37	46	44
Total courses/cycles	247/124	392/98	368/123	133/67
Weeks on study^a^	18.6	12.0	15.2	7.0
% cycles reduced	11	13	8	14
% cycles delayed	17	26	18	34
RDI^b^	0.96 (0.69–1.02)	0.97 (0.60–1.11)	0.99 (0.61–1.05)	0.90 (0.21–1.04)

a Median.

b Median (min–max).

. In arm A, the main reasons for treatment delay were vomiting and infection (two patients, two cycles); in arm B neutropenia (10 patients, 13 cycles); and in arms C and D diarrhoea (five patients, five cycles and six patients, eight cycles, respectively). Few cycles had dose reductions. The main reasons for dose reductions were diarrhoea in arm A (four patients, four cycles), arm C (five patients, five cycles) and arm D (seven patients, 10 cycles) and neutropenia in arm B (seven patients, seven cycles). More protocol-planned doses were administered in arms A, B and C compared with arm D, where the relative dose intensity (RDI) was the lowest.

### Efficacy

Table 4

**Table 4:** Response results in the intent-to-treat population

	**Arm A**		**Arm B**		**Arm C**		**Arm D**	
	******N******	**%**	******N******	**%**	******N******	**%**	******N******	**%**
Complete response	0	0	0	0	0	0	1	2
Partial response	3	7	2	5	5	11	1	2
Stable disease	18	44	23	62	25	54	19	43
Progressive disease	19	46	7	19	15	33	15	34
Not evaluable	1	2	5	14	1	2	8	18
								
Overall response (CR+PR)	3	7	2	5	5	11	2	5
95% confidence interval	1.5–19.9		0.7–18.2		3.6–23.6		0.6–15.5	

summarises the overall response rates of the full analysis population by treatment arm. There were no significant differences in response rate across the four treatment arms (*P*=0.7). Table 5

**Table 5:** Secondary efficacy parameters in the intent-to-treat population

	**Arm A**	**Arm B**	**Arm C**	**Arm D**
	**Months**	**Months**	**Months**	**Months**
Duration of response and stabilisation	5.9 (0.8–12.7)	3.9 (1.0–13.0)	5.3 (1.3–8.9)	3.4 (1.3–6.9)
Time to progression	2.7 (1.0–12.8)	3.5 (0.0–13.2)	3.8 (0.0–8.9)	2.8 (0.0–7.0)
Time to treatment failure	2.7 (1.0–12.8)	2.3 (0.6–8.3)	3.2 (0.4–7.1)	1.7 (0.5–7.1)
Survival	9.4 (1.0–15.3)	7.1 (0.6–13.5)	8.6 (0.7–16.7)	6.4 (1.1–15.1)

Data are represented as median (min–max).

summarises the secondary efficacy parameters. Again, no significant differences in duration of response and disease stabilisation, time to treatment failure, time to progression or survival were observed. However, a notably shorter time to treatment failure was observed for arm D (1.7 months; *P*=0.02).

### Safety

The number of patients with at least one grade 3–4 adverse event possibly or probably related to irinotecan administration was 12 (29%) in arm A, 11 (30%) in arm B, 14 (30%) in arm C and 22 (50%) in arm D (*P*=0.1). Table 6

**Table 6:** Haematologic toxicity

**Toxicity type**	**Arm A**	**Arm B**	**Arm C**	**Arm D**
	**%Patients/%Cycles**			
*Leukocytopenia*				
All grades	73/70^a^	68/53^a^	70/56^a^	30/24^b^
Grade 3–4	20/15^b^	14/6	15/6^b^	5/3^b^
				
*Neutropenia*				
All grades	73^a^/65^b^	61^a^/50	65^a^/57^b^	23^a^/15^b^
Grade 3–4	34/21	25/12	28/20	9/5
Febrile neutropenia/neutropenic infection	75	5/2	7/1	—
	2/1	—	2/1	2/1

*Anaemia*				
All grades	98/92	92/87	91/90	91/83
Grade 3–4	2/1	3/1	4/4	0/0

*Thrombocytopenia*				
All grades	24^c^/14	11^c^/6	15^c^/6	9^c^/8
Grade 3–4	0/0	0/0	0/0	5/3

Percentage of patients developing toxicity in all courses/percentage of cycles in which toxicity developed; worst grade during study. Number of patients is 41 in arm A, 37 in arm B, 46 in arm C and 44 in arm D; number of cycles is 127 in arm A, 98 in arm B, 130 in arm C and 76 in arm D.

a Arm D is significantly different from arms A, B and C (*P*⩽0.004).

b Arm D is significantly different from arms A and C (*P*⩽0.004).

c Arm A is significantly different from arms B, C and D (*P*⩽0.001).

provides a summary of haematologic toxicities. Haematologic toxicity consisted primarily of leukocytopenia and neutropenia, and was generally more pronounced in arms A, B and C compared with arm D. The duration of neutropenia (grade 3–4) was <8 days in 53% of episodes in arm A, 77% in arm B, 84% in arm C and 25% in arm D. The median number of days to grade 3–4 nadir was higher in arm D (16 days, range 14–19) compared with arm A (9 days, range 6–17), arm B (7 days, range 4–13) and arm C (7 days, range 6–17). The median time to recovery from grade 3–4 neutropenia was the shortest in arm D (4 days, range 4–4) compared with arm A (7 days, range 6–14), arm B (7 days, range 2–15) and arm C (7 days, range 1–12).

Table 7

**Table 7:** Nonhaematologic toxicity

	**Arm A**		**Arm B**		**Arm C**		**Arm D**	
	**All grades**	**Grade 3–4**	**All grades**	**Grade 3–4**	**All grades**	**Grade 3–4**	**All grades**	**Grade 3–4**
**Toxicity type**	**% of patients**							
*Gastrointestinal origin*								
Diarrhoea	88	10	76	24	74	13	77	25
Nausea	73	15	73	5	65	9	80	23
Vomiting	54	10	57	3	50	9	59	16
Gastrointestinal pain	17	2	16	3	17	2	23	2
Anorexia	12	—	14	—	17	—	34	5
Other								
Fatigue	51	7	38	3	61	4	52	7
Fever in absence of infection	12	2	19	-	15	4	16	—
Infection	7	2	11	5	11	—	11	7
Cholinergic syndrome	68^a^	—	41^a^	—	61^a^	2	7^a^	—
Alopecia	71^b^	—	43	—	67^b^	—	34^b^	—

Toxicity is expressed as % of patients with adverse events possibly or probably related to study drug medication; worst grade during study. *N*=41 in arm A, 37 in arm B, 46 in arm C and 44 in arm D.

a Arm D is significantly different from arm A, B and C (*P*⩽0.001).

b Arm D is significantly different from arm A and C (*P*<0.001).

provides a summary of observed nonhaematologic toxicities. The main nonhaematologic toxicities were of gastrointestinal origin and were observed in all treatment arms. There was a trend towards more pronounced gastrointestinal toxicity in arm D. Severe diarrhoea was more frequently reported in arms D (25.0%) and B (24.0%), but was unexpectedly low in arm A (10%). The low rate of grade 3–4 diarrhoea in arm A is the result of both better patient information and good compliance to guidelines on the management of diarrhoea. Severe nausea and vomiting were also more often noted in arm D (23 and 16%, respectively) than in the other arms. Severe fatigue was equally reported in arms A and D (7 and 7%, respectively). Grade 3–4 anorexia was only reported in arm D. Other nonhaematologic toxicities were generally mild (grade 1–2). The incidence of alopecia was significantly less in arm D compared with arms A and C (*P*<0.001), and cholinergic syndrome was significantly less common in arm D compared to the other arms (*P*<0.001).

### Pharmacokinetics

Pharmacokinetic parameters of irinotecan and SN-38 are presented in Table 8

**Table 8:** Pharmacokinetic parameters of irinotecan and SN-38

			**Irinotecan**			**SN-38**			
**Arm**	**Dose (mg m^−2^)**	***N***	**Cl (l h^−1^)**	***C*_max_ (*μ*mol l^−1^)**	**AUC (*μ*mol h l^−1^)**	***C*_max_ (*μ*mol l^−1^)**	**AUC (*μ*mol h l^−1^)**	**AUC_c_ (*μ*mol h l^−1^)**	**Metabolic ratio**
A	350	30	22.3±7.3	7.91±1.10^c^	47.8±19.0^c^	0.151±0.069^d^	2.08±0.96^d^	4.16±1.93^e^	0.044±0.008^e^
B	125	28	26.8±9.6	2.62±0.39^c^	14.6±6.9^c^	0.065±0.021^d^	0.76±0.29^d^	3.03±1.17^f^	0.054±0.014^e^
C	250	30	24.0±8.4	5.52±0.90^c^	32.7±13.5^c^	0.117±0.057^d^	1.48±0.71^d^	4.44±2.12^e^	0.046±0.010^e^
	140^a^	6	30.4±8.2	ND	12.8±3.9	ND	1.4±0.3	4.2±0.9	0.12±0.02^b^

Data are listed as mean±s.d.

a Data obtained in patients receiving 10 mg m^−2^ irinotecan as 14-day prolonged infusions (*N*=6).^16^

b Significantly different from arms A–C (*P*<0.001).

c Significantly different from the other arms of treatment (*P*<0.001).

d Significantly different from the other arms of treatment (*P*<0.05).

e Significantly different from arm B (*P*<0.05).

f Significantly different from arms A and C (*P*<0.05). Abbreviations: see Materials and methods section. ND=not determined.

. The different doses of irinotecan lead to significant differences in the AUC and *C*_max_ of irinotecan and SN-38 across arms A, B and C during the first course. To compare the three treatment arms, the AUC of SN-38 was extrapolated to AUC per cycle (AUC_c_). The AUC_c_ is significantly higher in arms A and C compared with arm B, which is in accordance with the higher dose administered per cycle in arms A and C. The metabolic ratio in arms A and C was lower compared with arm B. No significant difference in clearance of irinotecan could be observed between arms A, B and C. We found a significant correlation between the administered dose of irinotecan and the AUC and *C*_max_ of irinotecan (*r*=0.69 and 0.93, respectively) and the AUC and the *C*_max_ of SN-38 (*r*=0.59 and 0.55, respectively) (*P*<0.001). The metabolic ratio of SN-38 was significantly inversely correlated with dose (*r*=−0.39, *P*<0.001).

## DISCUSSION

Irinotecan monotherapy is recognised as the treatment of choice in second-line therapy (after failure of 5-FU) in metastatic colorectal cancer (Cunningham *et al*, 1998; Douillard *et al*, 2000). Several administration schedules have been developed in the past; this study was set out to find the optimal schedule. This is the first report of a randomised phase II study of irinotecan given at four different schedules to patients with metastatic colorectal cancer.

After analysing the primary and secondary end points, no significant superiority of one of the four treatment schedules could be observed; this may be because the sample size was too small. There is a trend for an increased response rate in arm C; however, this does not translate to better survival. In addition, the response rate in arm D is possibly biased because of the relative high number of nonevaluable patients, the relatively shorter duration of treatment and the lower RDI. Median survival in this study was the highest in arm A (9.4 months), which is comparable to the median survival reported by Cunningham *et al* (1998) (9.2 months) and Rougier *et al* (1997) (10.8 months) using the same schedule.

The main toxicities were of gastrointestinal origin for all four treatment arms and haematologic toxicity was generally mild. We found no significant differences in the intensity of toxicity; however, there was a distinct difference in toxicity profiles. There was a trend for increased gastrointestinal toxicity in arm D (prolonged infusion) compared with the other treatment arms (short infusions). Furthermore, there was significantly less alopecia in arm D compared with arms A and C, and cholinergic syndrome was significantly greater in arms A, B, and C compared with arm D. The alopecia and cholinergic syndromes are probably more closely related to peak concentrations of irinotecan and SN-38, than to total dose intensity. This is in accordance with the results of a phase I study with prolonged schedules of irinotecan, where no cholinergic syndrome was observed. It has been found that the observed cholinergic syndrome after the administration of irinotecan is caused by a rapid reversible inhibition of acetylcholinesterase by irinotecan (Rivory *et al*, 1996). Furthermore, support for schedule-dependent toxicity was recently given by Fuchs *et al* (2003). In this study, a similar schedule as given in treatment arm A was compared with 125 mg m^−2^ irinotecan weekly for 4 weeks, followed by 2 weeks rest. A significantly lower incidence of severe diarrhoea was reported, while efficacy was comparable for both treatment arms. In addition, Hwang *et al* reported significantly less diarrhoea when 125 mg m^−2^ irinotecan was administered once a week for 2 weeks, followed by a week rest *vs* ‘schedule B’. However, in this study, also significantly less nausea, vomiting and neutropenia were observed (Hwang *et al*, 2003). Regarding haematologic toxicities, leukocytopenia and neutropenia were generally significantly milder in arm D compared with the other treatment arms in our study. Thrombocytopenia was significantly higher in arm A compared with the other treatment arms. These differences in toxicity profile are probably related to the schedule of administration rather than the dose per cycle (highest in arm C) or the exposure to SN-38 per cycle (lowest in arm B). Schedule dependency of topo-isomerase I inhibitors, with regard to cytotoxicity, was also shown *in vitro* and *in vivo* in tumour-bearing mice and in clinical studies with different camptothecins (Burris *et al*, 1992; Dodds and Rivory, 1999; Gerrits *et al*, 1999). The results of an early phase II study with irinotecan support the use of prolonged exposure schedules in patients with lymphomas (Ohno *et al*, 1990). Schedule dependency of irinotecan in patients with solid tumours is less obvious (Takeuchi *et al*, 1991; Sakata *et al*, 1994). Tumour response and safety of irinotecan may not only be schedule dependent but also tumour-type dependent. Increased carboxylesterase activity has been found in some tumour types and might influence conversion of irinotecan to SN-38 (Wakui and Taguchi, 1992; Guichard *et al*, 1999). Systemic exposure to SN-38 was found to be higher in mice bearing human neuroblastoma xenografts as compared with nontumour-bearing mice (Guichard *et al*, 1999).

We compared our pharmacokinetic parameters with the pharmacokinetics of patients treated with a prolonged infusion of irinotecan as described by Herben *et al* (1999), which is similar to the schedule used in arm D. We found a significant difference in exposure to SN-38 as estimated by the AUC_c_. The exposure to SN-38 was increased in arms A and C compared with arm B. The estimated exposure per cycle to SN-38 of patients treated by 14-day prolonged infusion of 10 mg m^−2^ day^−1^ irinotecan was comparable with arms A and C (Table 8) (Herben *et al*, 1999). This difference in exposure did not lead to differences in activity between arm B and arms A and C, nor could it explain for the differences found in toxicity profile. The AUC and *C*_max_ were strongly correlated with the dose of irinotecan. The metabolic ratios of SN-38 after the short infusions in arms A, B and C found in this study were 0.04, 0.05 and 0.04, respectively. A similar metabolic ratio was also found in a previous study where a metabolic ratio of 0.05 was noted after a standard 90-min infusion of 350 mg m^−2^ irinotecan (Rougier *et al*, 1997). This is lower than the metabolic ratio of 0.12, as found for the prolonged infusion of irinotecan (Table 8) (Herben *et al*, 1999). The difference in metabolic ratio between bolus and prolonged infusions agrees with other previously published data (Chabot *et al*, 1995; Gupta *et al*, 1997; Zamboni *et al*, 1998). We also found a significant difference between the metabolic ratio of SN-38 between arms A and B, and B and C. A low, but very significant inverse correlation between dose and metabolic ratio of SN-38 was observed for arms A, B and C. A possible explanation for this change in metabolic ratio is saturation of the carboxylesterase reaction. The carboxylesterase reaction converting irinotecan into SN-38 in humans is very inefficient and deacylation dependent (Rivory *et al*, 1996; Haaz *et al*, 1997; Takimoto *et al*, 2000). The saturability of this enzymatic reaction may be of relevance for prolonged infusion schedules of irinotecan. The metabolic ratio of SN-38 is not solely dependent on the formation of SN-38 by carboxylesterases, but also on the effect of dose on the metabolism of irinotecan via other pathways, such as the formation of APC, the saturation of its conversion to SN-38 glucuronide or enterohepatic recycling. SN-38, the active metabolite of irinotecan, is about a 100–1000 times more potent topo-isomerase I inhibitor than its parent compound *in vitro* models (Slatter *et al*, 1997). The observed extensive metabolism of irinotecan to its active form, SN-38, may thus explain in part the low recommended dose for the continuous infusion schedule (Herben *et al*, 1999). The extent of metabolism of irinotecan to SN-38 seems to be dependent upon the administration schedule and administered dose of irinotecan. This effect has no clinical relevance with the administration schedules used in arms A, B and C (short infusions). However, it provides a rationale for prolonged administration schedules with low-dose irinotecan, despite the slightly less favourable toxicity profile of prolonged intravenously administrated irinotecan, as found in this study.

Recently, an oral formulation of irinotecan has been developed and has entered phase I clinical trials (Schoemaker *et al*, 2002). Since chronic infusions are cumbersome and expensive, an oral formulation provides an excellent method to administer irinotecan over a prolonged period at low doses. However, future studies should examine whether the balance between efficacy and safety is most optimal in chronic (oral) treatment schedules.

In summary, the four irinotecan schedules used in this study can generally be considered equivalent, in terms of efficacy and toxicity. However, more patients may have been required for us to observe any significant differences. The schedule used in arm D resulted in more gastrointestinal toxicity but the least haematologic toxicity. Prolonged intravenous administration of irinotecan (arm D) showed no clinical benefit over the short infusion schedules and is therefore not a feasible treatment option because of the disadvantages associated with this mode of administration. Irinotecan can be administered safely as a second-line treatment to patients with colorectal cancer according to local clinical practice, using either weekly, every 2-week or every 3-week short infusion schedules.
